# CsLAZY1 mediates shoot gravitropism and branch angle in tea plants (Camellia sinensis)

**DOI:** 10.1186/s12870-021-03044-z

**Published:** 2021-05-28

**Authors:** Xiaobo Xia, Xiaozeng Mi, Ling Jin, Rui Guo, Junyan Zhu, Hui Xie, Lu Liu, Yanlin An, Cao Zhang, Chaoling Wei, Shengrui Liu

**Affiliations:** State Key Laboratory of Tea Plant Biology and Utilization, Anhui Agricultural University, West 130 Changjiang Road, Hefei, 230036 Anhui China

**Keywords:** *CsLAZY1*, Branch angle, Tea plant, Gravitropism, Overexpression

## Abstract

**Background:**

Branch angle is a pivotal component of tea plant architecture. Tea plant architecture not only affects tea quality and yield but also influences the efficiency of automatic tea plant pruning. However, the molecular mechanism controlling the branch angle, which is an important aspect of plant architecture, is poorly understood in tea plants.

**Results:**

In the present study, three *CsLAZY* genes were identified from tea plant genome data through sequence homology analysis. Phylogenetic tree displayed that the *CsLAZY* genes had high sequence similarity with *LAZY* genes from other plant species, especially those in woody plants. The expression patterns of the three *CsLAZYs* were surveyed in eight tissues. We further verified the expression levels of the key *CsLAZY1* transcript in different tissues among eight tea cultivars and found that *CsLAZY1* was highly expressed in stem. Subcellular localization analysis showed that the CsLAZY1 protein was localized in the plasma membrane. *CsLAZY1* was transferred into *Arabidopsis thaliana* to investigate its potential role in regulating shoot development. Remarkably, the *CsLAZY1* overexpressed plants responded more effectively than the wild-type plants to a gravity inversion treatment under light and dark conditions. The results indicate that *CsLAZY1* plays an important role in regulating shoot gravitropism in tea plants.

**Conclusions:**

The results provide important evidence for understanding the functions of *CsLAZY1* in regulating shoot gravitropism and influencing the stem branch angle in tea plants. This report identifies *CsLAZY1* as a promising gene resource for the improvement of tea plant architecture.

**Supplementary Information:**

The online version contains supplementary material available at 10.1186/s12870-021-03044-z.

## Background

The tea plant (*Camellia sinensis*) is an economic crop of great importance. Its leaves can be used to produce most traditional caffeinated teas, which are the second most popular beverage worldwide [1, 2]. The productivity of tea plants is greatly affected by the architecture of tea plants. A well-designed tree architecture should minimize competition with adjacent crops for environmental resources, such as light. In densely planted stands, a relatively wide branch angle may help the plant escape some diseases by decreasing humidity, but it makes the plant occupy more space and increases the extent of shade [3]; therefore, optimal tree architecture may contribute to increasing the yield and yield stability of crops [4]. Tea plant architecture is affected by geometric and environmental factors as well as the tea estate plantation elevation; besides, plucking patterns including manual plucking, shear harvesting and monsoon seasons also affect tea plant architecture [5]. According to their branch angle degree, tea plants are classified into three plant architecture types: open, half open, and erect.

Plant architecture is significantly associated with plant hormones, including gibberellic acid (GA), auxin, cytokinin, and strigolactones (SLs). GA is thought to promote upward growth and inhibit bending, and it is highly likely to be responsible for the weeping trait [6, 7]. Previous studies also suggested that genes associated with auxin and ethylene probably play crucial roles in shoot elongation [8, 9]. Cytokinin was identified as an important phytohormone that regulates plant shoot branching, it is synthesized in the roots and then transported throughout the plant for the development of the entire plant [10]. SLs are a group of newly identified plant hormones that are essential for regulating the shoot branch/tiller angle; they can inhibit auxin biosynthesis and attenuate rice shoot gravitropism, mainly by decreasing the local indoleacetic acid (IAA) content [11].

Many environmental signals, including light and gravity, can influence plant architecture [6, 12]. The branch angle is an important factor in determining plant structure and is regulated by specific genes. To date, many genes and transcription factors associated with branch angle have been identified. For instance, overexpression of *OsPIN2* leads to increased tiller numbers, and altering *OsPIN2* expression through genetic transformation can be directly used to modify rice architecture [13]. *OsTAC1* controls tiller angle in rice [3], and changes in *TAC1* have since been linked to upright tiller or branch angles in other plant species, including *Arabidopsis* [14], rice [15], poplar [4], peach [16, 17], and apple [18]. *OsTAC4* participates in the regulation of rice tiller angle, and influences the endogenous auxin content, ultimately leading to reduced gravitropism and a tiller-spreading phenotype [19, 20].

In many plant species, *LAZY1* plays an important role in regulating the plant branch angle. For example, the rice *lazy1* mutant displays a tiller-spreading phenotype because gravitropism is reduced [21]. In *Arabidopsis*, a total of six *LAZY* genes have been identified, and mutating *AtLAZY1* caused a large change in branch angle while the primary inflorescence stem remained vertical [22]. The other *lazy* mutations reversed the growth angle of lateral branches and roots, indicating that *LAZY* genes regulate the direction of polar auxin transport in response to gravity through the control of asymmetric *PIN3* expression in the root cap columella [23]. In apple and poplar, evidence has shown that *LAZY* genes affected the vascular tissues of transgenic plants, thus modifying the branch angle [4, 16].

Although *LAZY* genes have been indicated to play an important role in modifying branch angle in a variety of plant species, the potential function of homologous genes in tea plants (*Camellia sinensis*) is still unknown. Branch angle is an important trait of tea plants that can influence the plant architecture as well as the mechanical harvesting of tea leaves. In this study, three *LAZY* genes were identified in the tea plant, and their expression levels in distinct tissues were characterized. *CsLAZY1* was expressed predominately in stem and was located in the plasma membrane. Plants that overexpressed *CsLAZY1* responded more effectively than the wild-type plants to gravity processing. Our results identify new candidate genes that can be used to breed new tea varieties with ideal plant architecture.

## Results

### Identification, conserved domain and sequence feature analysis of *CsLAZYs*

A total of six *LAZY* genes were identified in *Arabidopsis thaliana* [22]. Subsequently, these six *AtLAZY* genes were used as queries in Basic Local Alignment Search Tool (BLAST) analysis against the tea plant genome (http://tpia.teaplant.org/Blast.html) [24]. Initially, a total of 15 candidate unique genes were obtained for tea plants, and multiple sequence alignments of all *LAZY* genes were performed among tea plant, *Arabidopsis* and rice (data not shown). The results showed that only 3 unique genes contained regions of conserved sequence V that possess an ethylene-responsive element-binding factor-associated amphiphilic repression (EAR) motif (LxLxL) (Figure S1) and that this is an indispensable conserved domain of LAZY [22, 25]. Thereafter, the three obtained genes were referred to as *CsLAZY1* (CSS025254), *CsLAZY2* (CSS049138) and *CsLAZY3* (CSS020288), and they were located in different scaffolds (Table 1). Their amino acid lengths were 399 aa (*CsLAZY1*), 367 aa (*CsLAZY2*) and 251 aa (*CsLAZY3*), respectively. Furthermore, the molecular weights (Mw) of *CsLAZY1* to *CsLAZY3* were 44.2, 41.2 and 29.0, and their isoelectric points (pI) were 6.55, 6.18 and 6.47, respectively (Table 1).

**Table 1:** Characterization of CsLAZYs in tea plant

Gene name	Gene ID	Genomic position	CDs (bp)	ORF (aa)	MW (kDa)	pI
CsLAZY1	CSS025254	Scaffold308520-312,877	1200	399	44.2	6.55
CsLAZY2	CSS049138	Scaffold357075-360,635	1104	367	41.2	6.18
CsLAZY3	CSS020288	Scaffold190296-193,383	756	251	29	6.47

### Evolution and phylogenetic analysis of *LAZY* genes

**Fig. 1:**
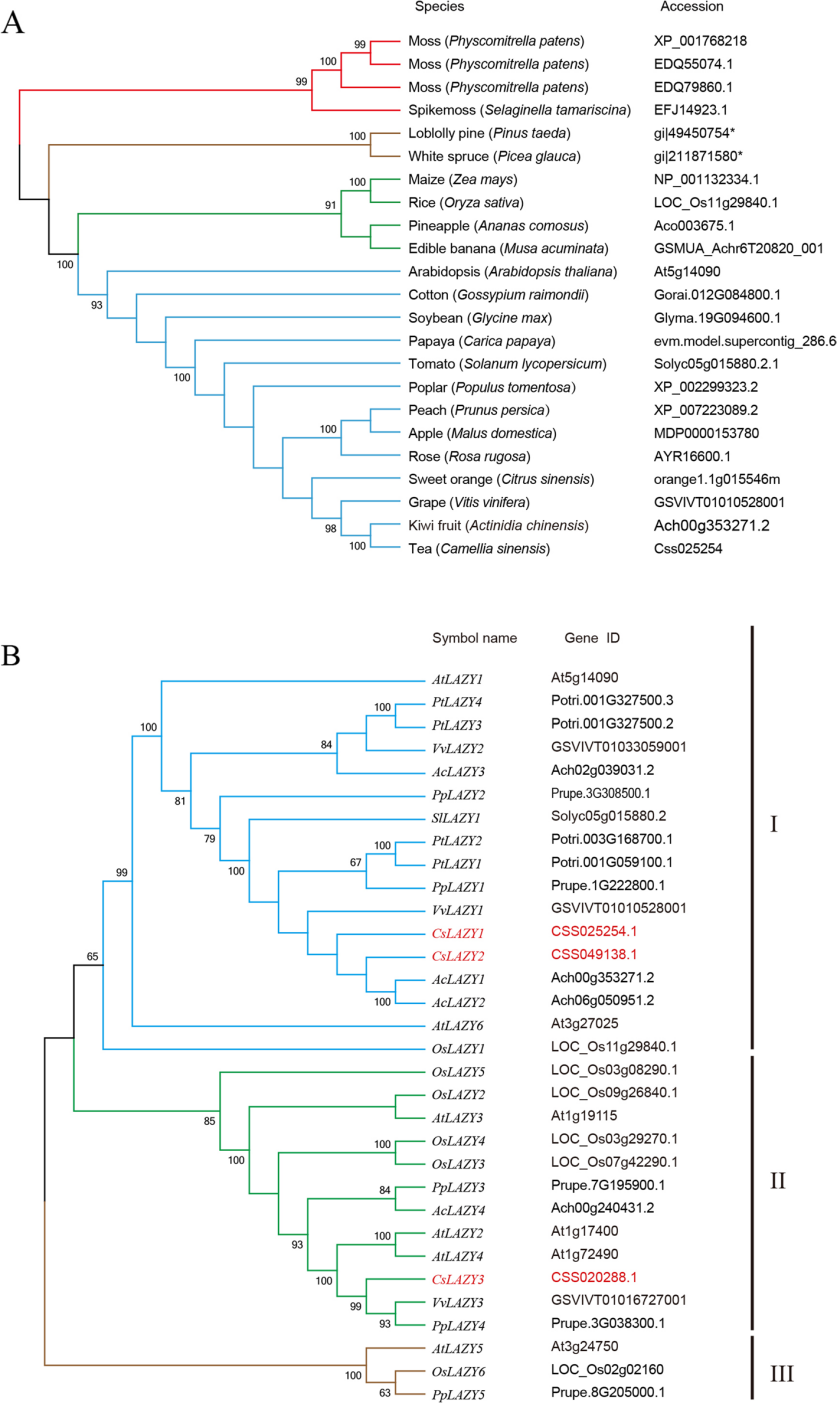
Phylogenetic relationships of *LAZY* genes from various plant species. **A** Phylogenetic tree of LAZY1 proteins identified from various plant species. Plant classifications are coded with different colours as shown in the legend, and the plant species and sequence accession IDs are listed. **B** Phylogenetic tree of the LAZY family protein sequences from eight plant species (*Os: Oryza sativa, At: Arabidopsis thaliana, Sl: Solanum lycopersicon, Pt: Populus tomentosa, Vv: Vitis vinifera*, *Pp: Prunus persica*, *Ac:* *Actinidia chinensis*, and *Cs: Camellia sinensis*). The phylogenetic trees were generated by MEGA 6.0 with the neighbor-joining algorithm with bootstrap analysis for 1000 replicates, and a p-distance model was used to ensure that the divergent domains could contribute to the topology of the NJ tree

Previous studies and the existence of numerous fully sequenced plant genomes have made it possible to perform a comparative genomic analysis of *LAZY* genes across a broad range of plant species. *LAZY* genes have been identified as playing similar roles in many plant species, so we performed iterative BLAST searches to determine the phylogeny of *LAZY1* genes. *LAZY1* genes were identified from 21 distinct plant species, and a homology analysis of *LAZY1* among algae, lowland species, monocots, and dicots provided further insight into the evolutionary processes of this gene family (Fig. 1A). Phylogenetic analyses showed that these *LAZY1* genes were highly conserved among algae, monocots and dicots and that *LAZY1* evolved from primitive organisms despite their overall relatively low sequence similarities. It was obvious that *CsLAZY1* had higher sequence similarity with *LAZY1* genes from other woody plants, including kiwifruit, grape, poplar and peach, indicating that *CsLAZY1* was more highly conserved in the process of evolution within woody plants (Fig. 1A).

To further understand their sequence homology and potential biological functions, we analyzed the evolutionary tree containing all *LAZY* gene family members from eight plant species, including *Oryza sativa*, *Arabidopsis thaliana*, *Solanum lycopersicon*, *Populus tomentosa*, *Vitis vinifera*, *Prunus persica*, *Actinidia chinensis* and *Camellia sinensis*. The complete *LAZY* gene families, including 32 members, were used for phylogenetic analysis. It was showed that these *LAZY* genes were mainly classified into three clades: class I, class II and class III (Fig. 1B). *CsLAZY1* and *CsLAZY2* were grouped into class I, and they both had high sequence similarities with the protein sequences of the *AcLAZY1*, *AcLAZY2* and *VvLAZY1* genes. *CsLAZY3* was grouped into class II and showed high sequence similarity with the *VvLAZY3*, *PpLAZY4*, *AtLAZY2* and *AtLAZY4* genes.

### Analysis of gene structures, cis-elements in promoters and tissue expression patterns of the three *CsLAZY* genes

**Fig. 2:**
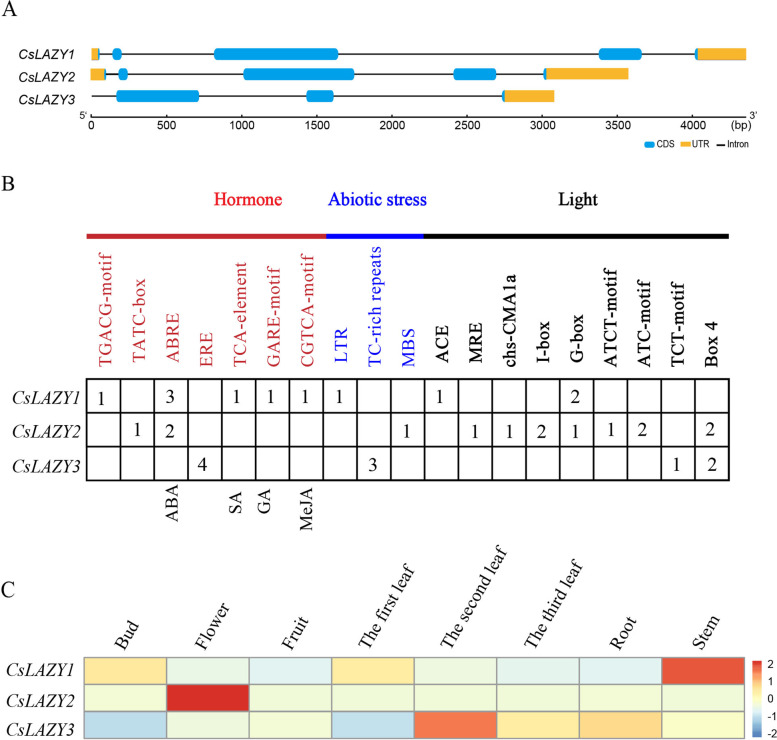
Analysis of gene structures, cis-elements in promoters and tissue expression patterns of the three *CsLAZY* genes. **A** Gene structures of the three *CsLAZY* genes from tea plants. **B** Prediction of cis-elements in the promoters of the three *CsLAZY* genes. **C** Expression patterns of the three *CsLAZY* genes in eight tissues

To investigate the structural diversity of *CsLAZY* genes, we compared the exon/intron organization in the coding sequences of each *CsLAZY* gene, demonstrating that *CsLAZY1* to *CsLAZY3* contained 5, 5, and 3 exons, respectively (Fig. 2A). In terms of intron and exon length, *CsLAZY1* was the longest while *CsLAZY3* was the shortest. The coding sequence of *CsLAZY1* was cloned and sequenced, demonstrating that the cloned cDNA was totally consistent with the genomic reference sequence.

To explore the potential differences in non-coding regions of the *CsLAZYs*, a 2-kb flanking sequence upstream of the translation start codon was obtained, and many putative cis-regulatory elements in the promoter were identified using the PLACE and PlantCARE databases (http://bioinformatics.psb.ugent.be/webtools/plantcare/html/). Light sensitive cis-elements, including Box 4, TCT-motif, ATC-motif, ATCT-motif, G-box, I-box, chs-CMA1a, MRE, and ACE, accounted for the largest proportion of all elements (Fig. 2B). *CsLAZY1* contained two kinds of key light-sensitive cis-elements, including two G boxes and one ACE; *CsLAZY2* contained seven kinds of light-sensitive cis-elements except for the ACE and TCT-motif elements. Seven kinds of hormone-sensitive cis-elements were obtained, including CGTCA-motif, GARE-motif, TCA-element, ERE, ABRE, TATC-box, and TGACG-motif. Notably, the promoter region of *CsLAZY1* contained five kinds of hormone-sensitive cis-elements, including TGACG-motif, ABRE, TCA-element, GARE-motif, and CGTCA-motif (Fig. 2B). These cis-elements are MeJA-, GA-, SA- and ABA-responsive elements, implying that *CsLAZY1* may play an important role in tea plant responses to hormones.

To understand the potential role of *CsLAZYs* in tea plants, we downloaded RNA-Seq data for eight tissues from the tea plant genome database. The data showed that the expression levels of the three *CsLAZY* genes were obviously specific to various tissues (Fig. 2C). For instance, *CsLAZY1* had the highest expression level in stem, followed by in bud and leaf, while it was basically not expressed in fruit or root. In comparison, *CsLAZY2* was expressed mainly in flower, and *CsLAZY3* showed the highest expression level in leaf. Because stem bending is one of the main causes of branch angle development, *CsLAZY1* probably plays a vital role in regulating the branch angle of tea plants.

### Expression patterns of *CsLAZY1* in tissues among different tea varieties

**Fig. 3:**
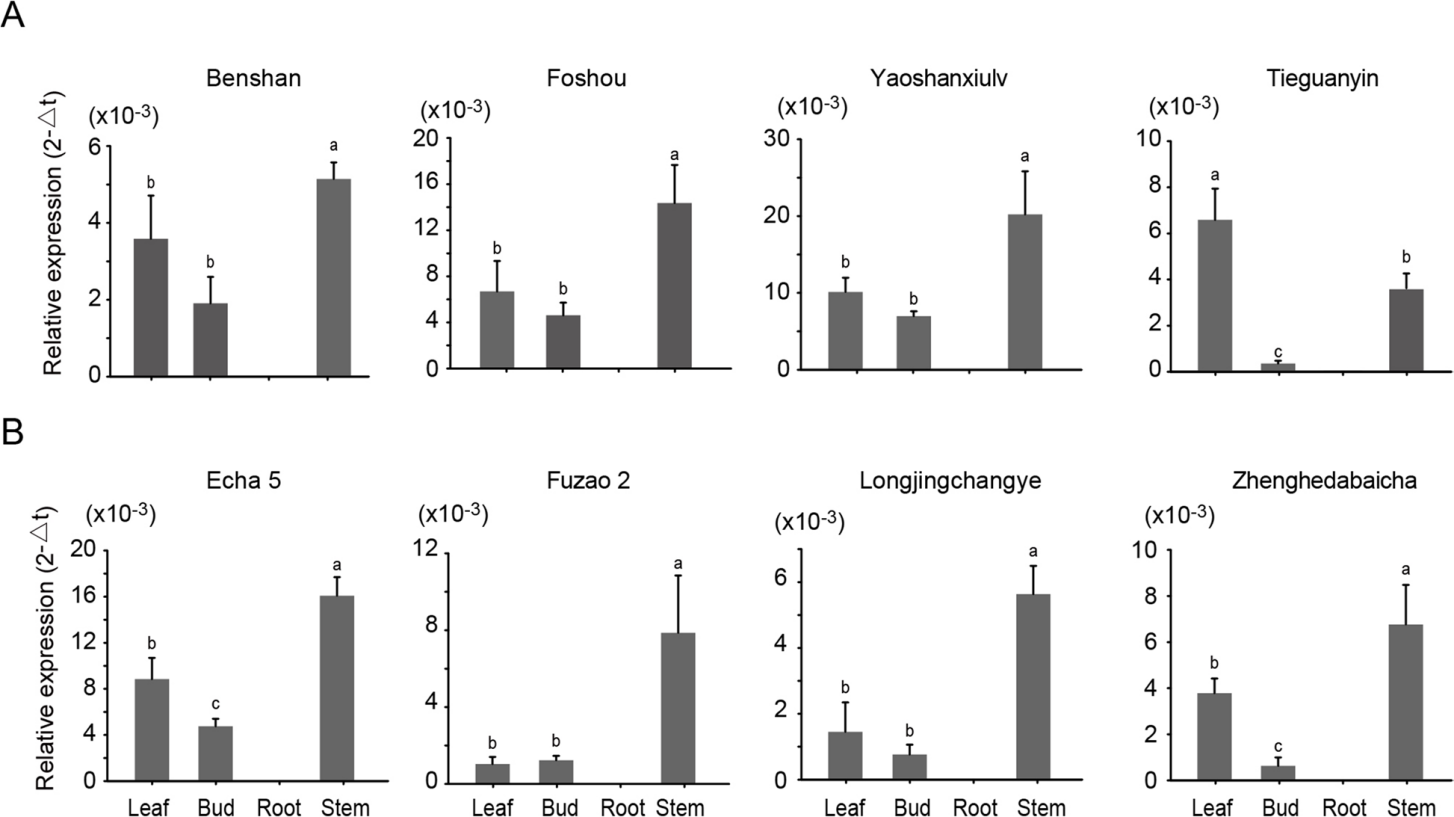
Expression patterns of *CsLAZY1* in four tissues of tea plants of two architecture types. **A** Tissue expression level of *CsLAZY1* in four open-type tea cultivars (Benshan, Foshou, Yaoshanxiulv, and Tieguanyin). **B** Tissue expression level of *CsLAZY1* in four erect-type tea cultivars (Echa 5, Fuzao 2, Longjingchangye, and Zhenghedabaicha). The expression levels of *CsLAZY1* in eight cultivars were analyzed by qRT-PCR. Bars indicate the means ± SDs (*n* = 3) of three biological replicates. Different letters above the bars denote significantly different levels of expression determined by Duncan’s multiple range test (*p* < 0.05)

To further verify the tissue expression pattern of *CsLAZY1*, we examined the tissue expression level of *CsLAZY1* in different tea varieties. A total of eight tea varieties with different branch angles, including four open-type varieties (Benshan, Foshou, Yaoshanxiulv, and Tieguanyin) and four erect-type varieties (Echa 5, Fuzao 2, Longjingchangye, and Zhenghedabaicha), were analysed. The expression level of the *CsLAZY1* transcript varied significantly among the four tissues (leaf, bud, root and stem) (Fig. 3). It was showed that *CsLAZY1* transcript was not detected in root of the eight tea varieties, and it had the highest expression level in stem, followed by that in leaf. Notably, no obvious difference in the tissue expression pattern was observed between the two different type tea plants. Unexpectedly, in the Tieguanyin cultivar, *CsLAZY1* had the highest expression level in leaf, followed by that in stem.

### Subcellular localization of CsLAZY1 protein

**Fig. 4:**
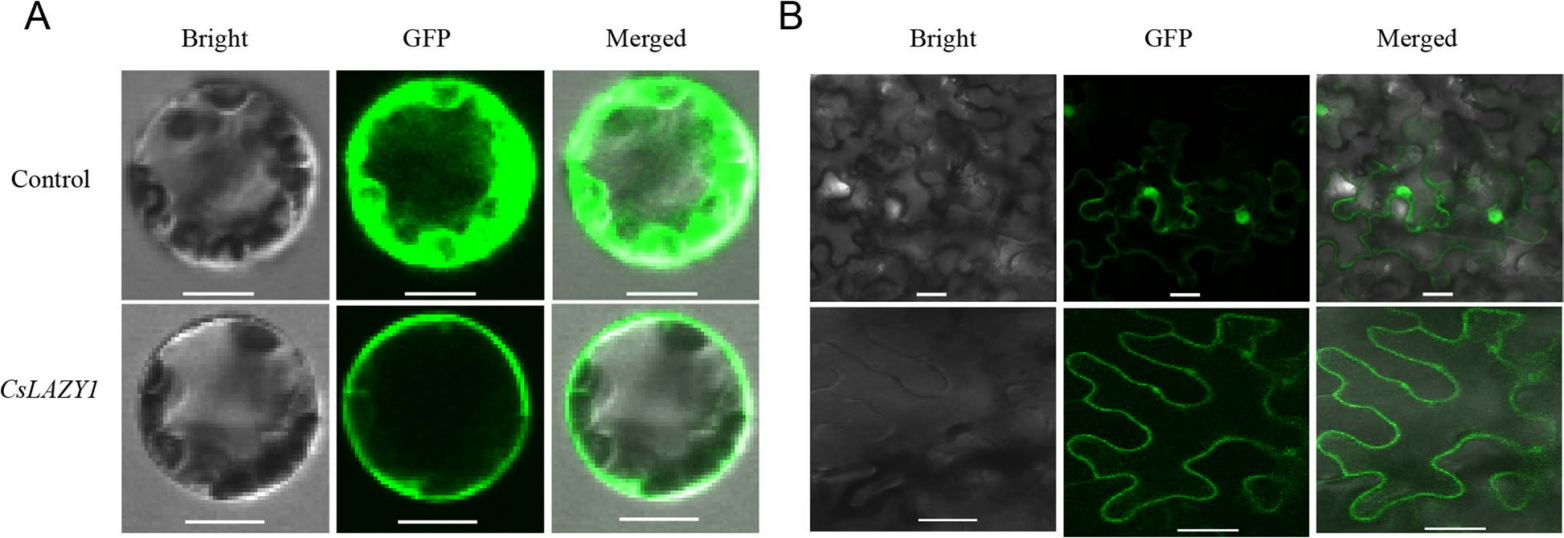
Subcellular localization of *CsLAZY1.***A** pk7WGF2 (empty vector) and pk7WGF2-CsLAZY1 were transformed into *Arabidopsis* protoplast cells. Scale bar = 10 μm. **B** pk7WGF2 (empty vector) and pk7WGF2-CsLAZY1 were transiently expressed in *Nicotiana benthamiana* leaves. Scale bar = 20 μm

In *Arabidopsis*, AtLAZY1, which contributes to the regulation of branch angles, is localized in the plasma membrane and nucleus [22]. To obtain insight into the molecular function of the CsLAZY1 protein, we constructed CsLAZY1-GFP and Pk7WGF2 35S-GFP fusion protein expression vectors to examine its subcellular localization. Transient expression in *Arabidopsis* protoplasts indicated that the CsLAZY1 protein was localized in the plasma membrane (Fig. 4A). In addition, the plasmid of CsLAZY1-GFP was transferred into *Agrobacterium* to infect *Nicotiana benthamiana* leaves and obtained identical results, namely, the CsLAZY1 protein was localized in the plasma membrane (Fig. 4B).

### Overexpression of *CsLAZY1* in *Arabidopsis*

**Fig. 5:**
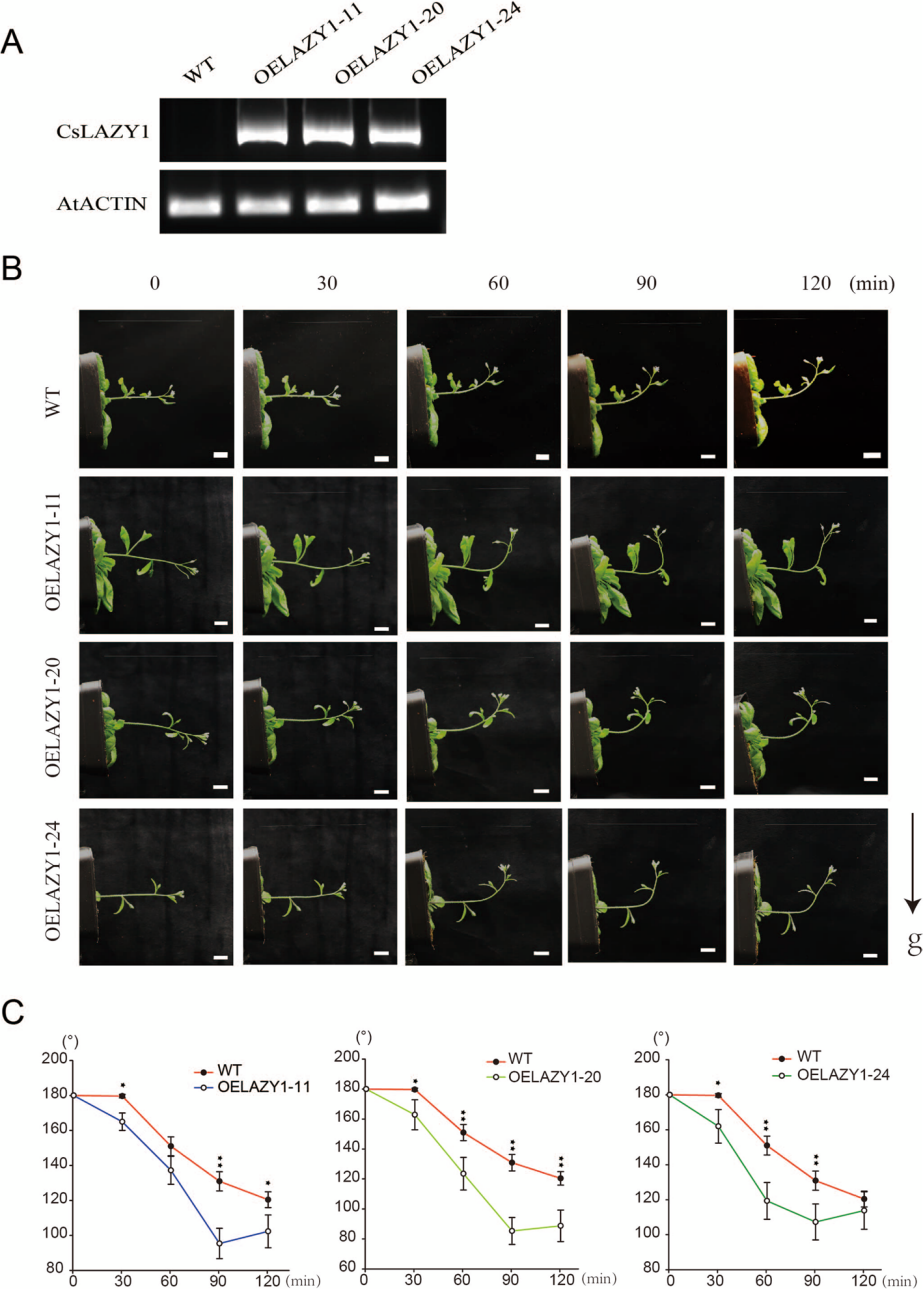
Gravitropic responses in wild-type (WT) and *CsLAZY1* overexpression (OE) *A.thaliana* in the light. **A** Expression level of *CsLAZY1* in WT and OE plants, the *AtACTIN* gene was used as the control. **B** WT and OE plants were treated at 90° inverted for 0, 30, 60, 90 and 120 min. Scale bar = 1 cm. **C** Bending angles of WT and OE plants. Bars indicate the means ± SDs (*n* = 3), and the asterisk above the bars denotes a significantly difference in bending angle determined by Duncan’s multiple range test (*P < 0.05, **P < 0.01)

To further investigate the role of *CsLAZY1* in shoot gravitropism, we transferred *CsLAZY1* into *Arabidopsis thaliana.* The expression of *CsLAZY1* was detected using a real-time PCR assay in *CsLAZY1*-overexpression (OE) plants but not in wild-type (WT) plants, and the three OE lines were named OELAZY1-11, OELAZY1-20, and OELAZY1-24 (Fig. 5A). Subsequently, gravitropism assays recorded with time-lapse imaging were implemented to survey the responses of the WT and three OE lines to reorientation. All seedlings with a main stem of 5–10 cm were subjected to 90° inverted gravity processing. In the light, images were collected by computer-controlled cameras after 0, 30, 60, 90 and 120 min of inversion (Fig. 5B), and the angle of the hypocotyls was measured from the images. The OE plants clearly bent upward slightly at 30 min, while no bending was observed in the WT plants. After 90 min of inversion, the OE plants reached their maximum bending angles, while the WT plants bent upward slightly (Fig. 5B). After 30 min treatment, a significant difference in bending angle was observed between the OE and WT plants (Fig. 5C).

**Fig. 6:**
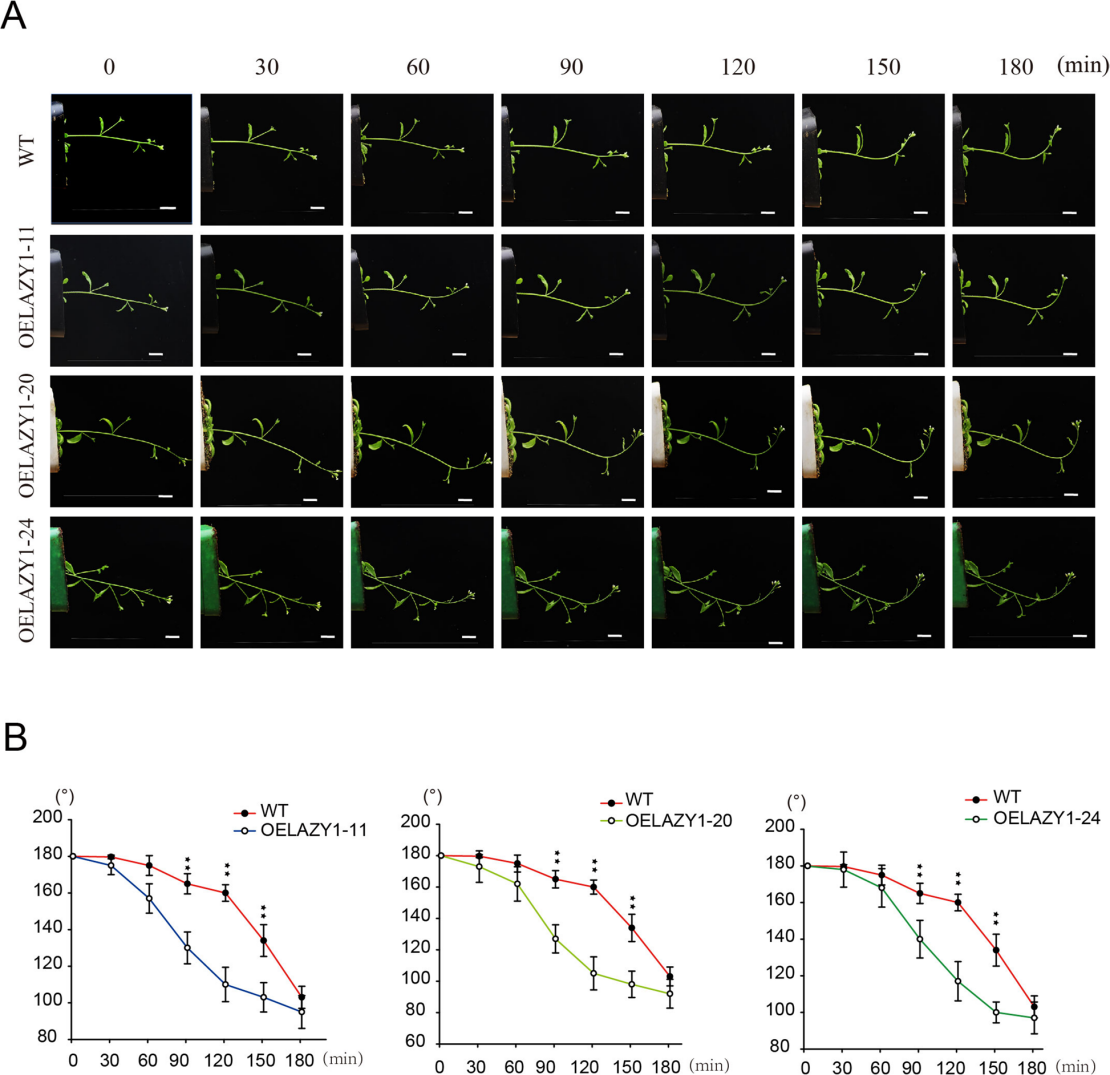
Gravitropic responses in wild-type (WT) and *CsLAZY1* overexpression (OE) *A.thaliana* in the dark. **A** WT and OE plants were subjected to 90°inversion for 0, 30, 60, 90, 120, 150 and 180 min. Scale bar = 1 cm. **B** Bending angles of WT and OE plants. Bars indicate the means ± SDs (*n* = 3), and the asterisk above the bars denotes a significantly difference in bending angle determined by Duncan’s multiple range test (*P < 0.05, **P < 0.01)

In the dark, images were collected after 0, 30, 60, 90, 120, 150 and 180 min of inversion, and the angle of the hypocotyls was measured from the images. Both the WT and OE plants bent upward later in the dark than their corresponding plants in the light, implying that the light-sensitive cis-elements in the promoter may be associated with the function of the *CsLAZY1* gene (Fig. 6A). In the dark, no bending angle was observed in the WT and OE plants after 30 min. The OE plants bent upward slightly after 60 min, while the WT plants bent upward slightly after 90 min (Fig. 6A). After 90 min, a significant difference in the bending angle was also observed between OE plants and WT plants (Fig. 6B). Consistent with the expression patterns, the evidence indicates that *CsLAZY1* may play a vital role in the response to gravitropism in stem of tea plants.

## Discussion

Agricultural productivity is affected by various environmental factors that can result in lower crop yields. Plant architecture is one of the major constraints on crop yield, and branch angle plays a vital role in the formation of plant architecture [26]. Accumulated evidence indicates that *LAZY1* plays a crucial role in plant responses to gravitropism and then regulates branch angles [22, 23, 27]. For tea plants, the branch angle is a critical factor that can greatly influence the productivity and efficiency of mechanical plucking. Nevertheless, the molecular mechanism controlling the branch angle of tea plants has scarcely been understood until now. In the present study, we identified three *CsLAZY* genes in tea plants and analyzed their phylogenetic relationships, gene structures and tissue-specific expression patterns. Subsequently, the biological function of the candidate gene *CsLAZY1* was investigated, including its subcellular localization, tissue-specific expression patterns in different tea varieties, and heterologous overexpression analysis were performed, which revealed obvious differential responses to gravity.

The three *CsLAZY* genes exhibited different tissue expression patterns (Fig. 2C). *CsLAZY3* was distinguished from the other two *CsLAZY* genes by clustering into a different subclade, which had a high expression level specifically in the second leaf. *CsLAZY2* showed high sequence similarity with *CsLAZY1* and had the highest expression level in flower, indicating that *CsLAZY2* may play an important role in the development of flower. In comparison, *CsLAZY1* had a far higher expression level in stem than in the other tissues (Fig. 2C), and similar tissue-specific expression patterns were observed in several other woody plants, such as poplar [4], peach [16] and apple [18]. To identify whether *CsLAZY1* exhibits different tissue-specific expression patterns in different tea varieties, we examined its expression in two types of tea plants, open-type plants (Benshan, Foshou, Yaoshanxiulv and Tieguanyin) and erect-type plants (Echa 5, Fuzao 2, Longjingchangye, Zhenghedabaicha), categorized based on their branch angle. No obvious difference in the expression pattern was observed among the different tea varieties (Fig. 3). In poplar, the transcript level of *PtLAZY1* showed similar expression profiles in different tissues, and no significant difference in tissue-specific expression profiles was observed between narrow-crown and broad-crown poplars [4]. In *Arabidopsis*, disruption of *AtLAZY1* expression can cause the weakening of gravitropic response, and lead to branch angles to become larger [22, 28], thereby the results accounted for the similar expression patterns of *CsLAZY1* in different tea varieties (Fig. 3). In addition, subcellular localization of *CsLAZY1* was analyzed, demonstrating that the CsLAZY1 protein is located in the plasma membrane, which is consistent with previous studies in *Arabidopsis* [22, 23, 28]. Additionally, several other *AtLAZY* genes from *Arabidopsis thaliana* are localized in the plasma membrane [22, 28]. Unexpectedly, it has been confirmed that OsLAZY1 is located in the nucleus and that the nuclear localization of OsLAZY1 is essential for its function in rice [29, 30]. In fact, the OsBRXL4 protein interacts with OsLAZY1 at the plasma membrane, and their interaction determines the nuclear localization of OsLAZY1, thereby OsBRXL4 regulates shoot gravitropism and the rice tiller angle by affecting the nuclear localization of LAZY1 [30]. Thereby, the majority of LAZY1 from different plant species are localized in the plasma membrane, only a little difference is existed of OsLAZY1 localization in rice.

*LAZY* genes that share common domain sequences usually have a common origin and thus have similar functions [22, 25]. The homology analysis of *LAZY-like* genes in poplar and the functional investigation of *PzLAZY* suggested that *PzLAZY* may be involved in altering branch angle [4]. Among *LAZY* genes, an EAR motif is located in conserved region V, which plays a role in controlling hormonal systems and is related to the gravitropic response of plants. For instance, the binding of TOPLESS proteins to the EAR motif of AUX/IAA proteins can repress auxin-responsive genes [30, 31]. The three *CsLAZY* genes obtained from tea plant shared five limited sequence regions and were highly conserved in region V (Figure S1), indicating that they may play a role in the development of tea plants. Phylogenetic analysis showed that *CsLAZY1* had high sequence similarity with *LAZY1* from other woody plants, including those in grape, poplar and peach (Fig. 1A). This result indicates that *LAZY1* experienced greater conservation in the process of its evolution within woody plants.

Furthermore, we obtained heterologous OE *Arabidopsis* plants, while no difference in phenotypes was observed between OE plants and wild type plants, which are also consistent with the results of a previous study [32]. Notably, the phenotype of the OE plants was obviously distinct from that of the wild types in response to gravity (Figs. 5 and 6). It was speculated that *CsLAZY1* may play roles in altering branch angle by acting on the transportation of phytohormones. In *Arabidopsis thaliana*, six *AtLAZY* genes participate in early gravity signalling for shoot gravitropism [22, 23, 28]. *AtLAZY1* leads to the asymmetric distribution of auxin, thus altering the rice tiller angle; *AtLAZY1* also mediates gravity signalling in statocytes downstream of amyloplast displacement, leading to the development of asymmetric auxin distribution in gravity responsive organs [33–35]. In rice, *OsLAZY1* controls the tiller angle by regulating shoot gravitropism through the inhibition of polar auxin transport [21, 29, 31]. We also analyzed the cis-elements in the promoters of *CsLAZYs* and found that MeJA, GA, SA and three ABA hormone-responsive elements existed in the promoter of *CsLAZY1* (Fig. 2B). Collectively, *CsLAZY1* may play roles in the response to gravitropism and alter the branch angle by acting on the transportation of phytohormones.

## Conclusions

In this study, we identified three *LAZY* genes from tea plants and named them *CsLAZY1* to *CsLAZY3* based on their sequence similarity with the *LAZY* genes from *Arabidopsis*. The *CsLAZY1* to *CsLAZY3* genes showed distinct expression patterns in eight different tissues, and had the highest expression levels in stem, flower and leaf, respectively. Tissue-specific expression of *CsLAZY1* was also identified in different tea varieties that exhibited distinct branch angles, confirming that *CsLAZY1* had the highest expression level in the stem. The CsLAZY1 protein was localized in the plasma membrane based on a subcellular localization analysis. The overexpression of *CsLAZY1* in *Arabidopsis thaliana* showed that overexpress plants responded more effectively than the wild type plants to gravity processing under light and dark conditions. The results indicated that *CsLAZY1* plays an important role in regulating shoot gravitropism and affecting the branch angle in tea plants.

## Materials and methods

### Plant materials

A total of nine five-year-old tea plant cultivars (*Camellia sinensis* var. ‘Shuchazao’, ‘Benshan’, ‘Foshou’, ‘Yaoshanxiulv’, ‘Tieguanyin’, ‘Echa 5’, ‘Fuzao 2’, ‘Longjingchangye’, and ‘Zhenghedabaicha’) from the Tea Plant Cultivar and Germplasm Resource Garden in Guohe town (Anhui Agricultural University) were used for the collection of various tissues (the second leaf, apical bud, young root, and young stem). All tissues were sampled according to the demands of each experiment, and they were immediately frozen in liquid nitrogen and stored at -80 °C until utilization.

### Identification and molecular cloning of *CsLAZYs*

The nucleotide and deduced amino acid sequences of 6 *AtLAZY* genes from *Arabidopsis* were obtained from TAIR (The Arabidopsis Information Resource) database (https://www.arabidopsis.org/). A genome-wide search of 6 *AtLAZY* genes was carried out using Basic Local Alignment Search Tool (BLAST) analysis with the 6 *AtLAZY* genes used as queries against the tea plant genome (http://tpia.teaplant.org/Blast.html) [36]. All nonredundant protein sequences were compared with AtLAZYs and OsLAZYs, and the genes possessing pivotal conserved domains were selected. To verify the coding regions of *CsLAZYs*, gene-specific primers were designed for the amplification of *CsLAZY* genes with cDNA templates from the young leaves of *Camellia sinensis* var. ‘Shuchazao’.

### Phylogenetic analysis of *LAZY *genes

Gene sequences from rice, tomato, apple, peach, poplar, and grape were obtained from Phytozome (https://phytozome.jgi.doe.gov/pz/portal.html), and gene sequences from kiwifruit were obtained from the Kiwifruit Genome Database (http://kiwifruitgenome.org/). Multiple sequence alignment of *LAZY* protein sequences was performed using the ClustalW program. Phylogenetic trees were generated based on the full-length amino acid sequences by MEGA 6.0 with the neighbor-joining (NJ) algorithm. Bootstrap analysis with 1000 replicates was used to evaluate the significance of the nodes, and a p-distance model was used to ensure that the divergent domains could contribute to the topology of the NJ tree.

### Gene structure and promoter structure analysis of *CsLAZY* genes

Alignment of amino acid sequences was performed using T-COFFEE (http://tcoffee.org/) [37]. Based on the gene structure display server (GSDS 2.0, http://gsds.cbi.pku.edu.cn/index.php) program, we determined the exon/intron organization of *CsLAZYs* by comparing the coding sequences to their corresponding genomic sequences. To investigate cis-elements in the promoter sequences of the *CsLAZY* family genes, a 2 kb flanking sequence upstream of the translation start codon were isolated, and the PLACE and PlantCARE (http://bioinformatics.psb.ugent.be/webtools/plantcare/html/) were used to identify cis-regulatory elements in the promoters.

### RNA extraction and real-time quantitative PCR analysis

Total RNA was extracted from tea leaves using the RNAprep Pure Plant Kit (cat DP432, Tiangen, Beijing) according to the manufacturer’s protocol. The quality and quantity of each RNA extract were detected using agarose gel electrophoresis and a Nanodrop 2000 (Thermo Fisher Scientific, US). First-strand cDNA was synthesized from total RNA using the PrimeScript RT Reagent Kit (cat RR036A, Takara, Japan) following the manufacturer’s protocol. A 10 μl total reaction volume, including 5 μl TB Green Enzyme, 1.2 μl cDNA, 3.2 μl water and 0.6 μl primer, was used for qRT-PCR, and the process was performed as described previously in detail [38, 39]. The *CsGAPDH* gene was selected as the internal control, and the relative gene expression values were analyzed using the 2^−△Ct^ method [40]. All reactions were run with triplicate technical replicates for each sample, and three biological replicates were performed. The relevant primers are listed in Additional file 1, and the full length nucleotide and protein sequences of *CsLAZY1* are listed in Additional file 2.

### Subcellular localization of CsLAZY1 protein

The *CsLAZY1* plasmid fused with GFP was constructed by Gateway Technology, and the ORFs of *CsLAZY1* with a 25 bp vector adapter were amplified by RT-PCR. PCR products were inserted into the pDONR207 vector by BP clone enzyme mix, and then transferred into PK7WGF2 through LR reactions. The resultant empty vector and pk7WGF2-LAZY1 plasmids were transformed into *Arabidopsis* protoplast cells, and the protoplasts were examined after transformation overnight. Besides, the resultant vectors were also transformed into *Agrobacterium* GV3101 competent cells, and the construct and empty vector were transiently introduced into *Nicotiana benthamiana* leaves by injection. The tobacco leaves were held for 48 h at 25 °C in the dark after transformation, and the tobacco leaves and protoplasts were examined using an Olympus FV1000 confocal microscope (Olympus, Japan).

### *Arabidopsis* transformation and branch angle measurements

The full-length cDNA sequences were ligated into PBI121 driven by CaMV35S and then transferred into Agrobacterium strain GV3101. *Arabidopsis* (Col) was transformed using the floral dip method as described previously [41]. Transformed plants were selected on the basis of their resistance to kanamycin, and 4-week-old homozygous T3 plants were used for further experiments. Three transgenic lines were subjected to 90° inverted gravity processing for analysis of the bending angle.

The plant response to the gravity angle was determined as follows: the stem was initially positioned in a standard alignment to allow angle changes at each time point to be detected, a tangent line was drawn along the initial stem and along the curved stem, and the angle between the two tangent lines was measured using IMAGEJ software.

## Supplementary Information

**Additional file 1**. The relevant primers

**Additional file 2**. Gene sequences of CsLAZY1

**Additional file 3: Figure S1**. Sequence alignment of five conserved regions of all LAZY family genes from rice, Arabidopsis and tea plants. All the LAZY genes were finally confirmed based on the EAR motif.

**Additional file 4: Figure S2**. Expression analysis of AtLAZY1 and CsLAZY1 in the WT and overexpression Arabidopsis plants.

## Data Availability

The data sets supporting the results of this article are available at the NCBI SRA database (https://www.ncbi.nlm.nih.gov/) under project accession number MW848488.

## Abbreviations

OE: Overexpression
WT: Wild type
qRT-PCR: Quantitative real-time polymerase chain reaction
IAA: Indoleacetic acid
ABA: Abscisic acid
SA: Salicylic acid
GA: Gibberellin
MeJA: Methyl Jasmonate
SLs: Strigolactones
BRXL4: Brevis Radix-like 4
PIN: PIN-FORMED
