# Supplementary figures and images for: CsLAZY1 mediates shoot gravitropism and branch angle in tea plants (Camellia sinensis)
Source: BMC Plant Biol. 2021 May 28;21:243. doi: 10.1186/s12870-021-03044-z (PMC8164267; doi:10.1186/s12870-021-03044-z)

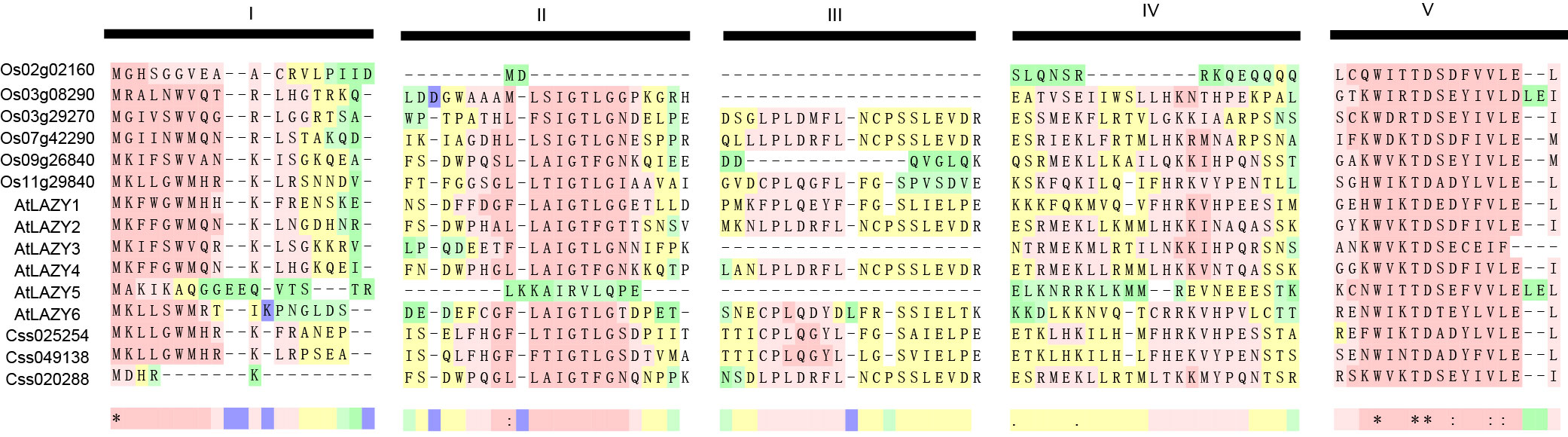

Supplement: Supplementary file 3 — Additional file 3: Figure S1. Sequence alignment of five conserved regions of all LAZY family genes from rice, Arabidopsis and tea plants. All the LAZY genes were finally confirmed based on the EAR motif. [file 12870_2021_3044_MOESM3_ESM.jpg]

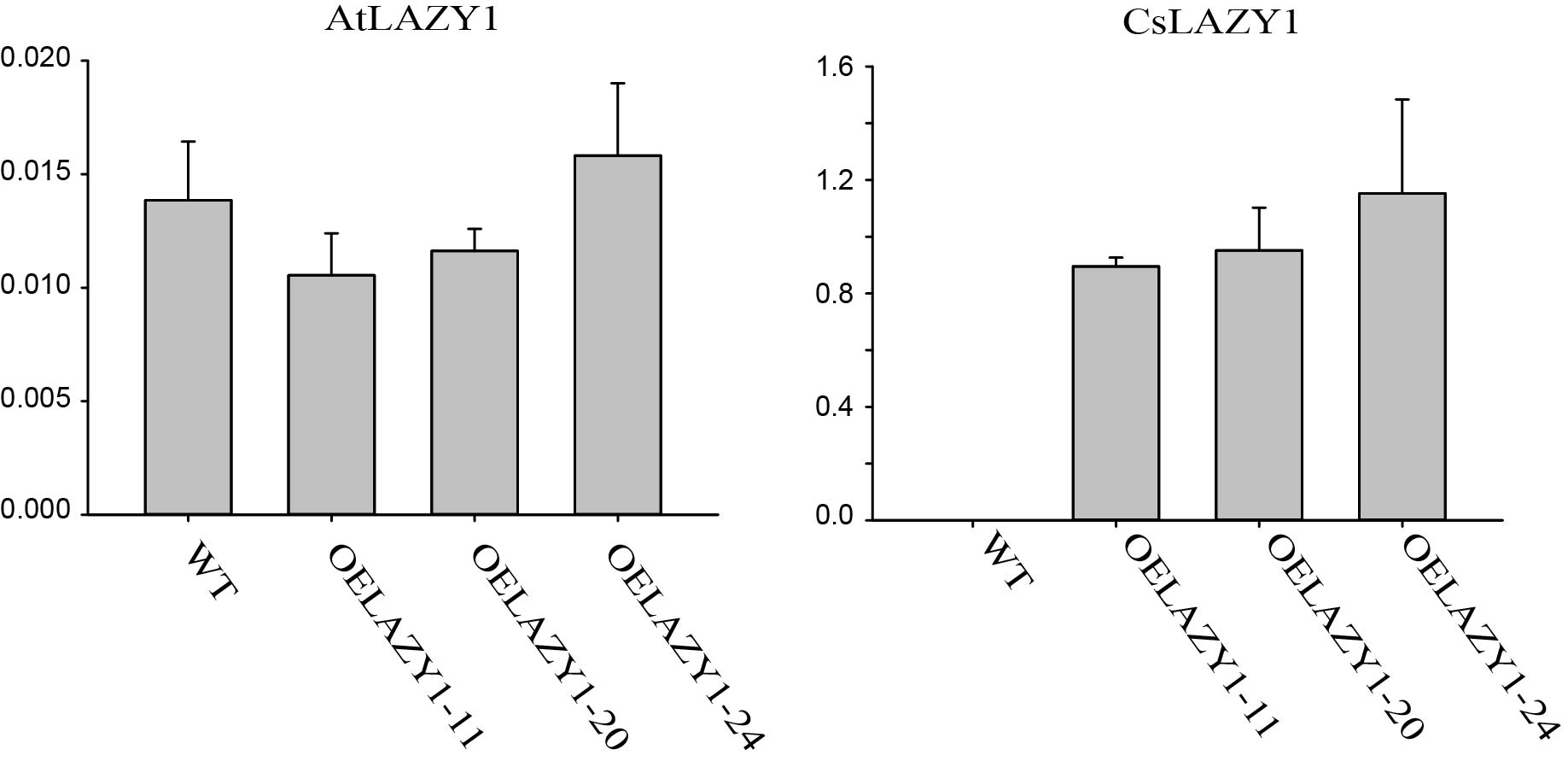

Supplement: Supplementary file 4 — Additional file 4: Figure S2. Expression analysis of AtLAZY1 and CsLAZY1 in the WT and overexpression Arabidopsis plants. [file 12870_2021_3044_MOESM4_ESM.jpg]
